# Translocation of intracellular CD24 constitutes a triggering event for drug resistance in breast cancer

**DOI:** 10.1038/s41598-021-96449-7

**Published:** 2021-08-23

**Authors:** Hugo Werner Huth, Thiago Castro-Gomes, Alfredo Miranda de Goes, Catherine Ropert

**Affiliations:** 1 Departamento de Biologia Celular, Universidade Federal de Minas Gerais, Belo Horizonte, Minas Gerais 31270-910 Brazil; 2 Departamento de Parasitologia, Universidade Federal de Minas Gerais, Belo Horizonte, Minas Gerais 31270-910 Brazil; 3 Departamento de Bioquímica e Imunologia, Universidade Federal de Minas Gerais, Belo Horizonte, Minas Gerais 31270-910 Brazil

**Keywords:** Cancer, Cell biology, Biomarkers, Molecular medicine, Oncology

## Abstract

The capacity of tumor cells to shift dynamically between different states could be responsible for chemoresistance and has been commonly linked to the acquisition of stem cell properties. Here, we have evaluated the phenotype switching associated with drug resistance in breast cancer cell lines and cell lineage obtained from Brazilian patients. We have highlighted the role of the cancer stem cell marker CD24 in the dynamics of cell plasticity and the acquirement of drug resistance. We showed that the translocation of CD24 from cytosol to cell membrane is a triggering event for the phenotype change of breast tumor cells exposed to drug stress. Here, we provide evidence that the phenotype switching is due to the presence of a cytosolic pool of CD24. Importantly, the cellular localization of CD24 was correlated with the changes in the dynamics of p38 MAPK activation. A strong and continuous phosphorylation of the p38 MAPK led to the overexpression of Bcl-2 after treatment in persistent cells presenting high density of CD24 on cell membrane. This phenotype enabled the cells to enter in slow-down of cell cycle, after which several weeks later, the dormant cells proliferated again. Importantly, the use of a p38 activity inhibitor sensitized cells to drug treatment and avoided chemoresistance.

## Introduction

Drug resistance continues to be the main limiting factor to cure patients with cancer that can be imputed to the adaptation capacity of tumor cells that corresponds to the emergence of resistant cells after anti-tumor therapy. Indeed, this phenomenon has been commonly associated with stemness when the acquisition of Cancer Stem Cell (CSC) properties by non-CSCs has been reported under several conditions, including drug treatment. CSCs would be responsible for tumor initiation, maintenance, metastasis formation, phenotypic plasticity and drug resistance^1–7^. CD24, a cell surface adhesion glycoprotein, was identified as a CSC marker and is present in various types of cancer including breast, pancreatic and lung^8^ and has often been associated with more aggressive diseases in ovarian, breast, lung and prostate cancers^9,10^. This explains the considerable interest for this marker in tumor biology and also in treatment outcome. In this line, relevant studies have revealed that the phenotype switching associated with the detection of surface CD24 could be responsible for chemoresistance^11,12^. For instance, CD24 expression level has appeared to be a significant molecular phenotype of cisplatin-resistant residual cells in laryngeal carcinoma lines, which corresponds to a differential expression of critical apoptotic and drug resistance^13^. Goldman et al. have observed an enrichment of CD24^+^ cells following treatment with docetaxel in different breast tumor cell lineages, which corresponded to the generation of new CSCs from non-CSCs^12^. Importantly, the mechanism leading to an increased surface CD24 expression in tumor cells under drug stress remains unknown.

Recent findings support the hypothesis that epigenetic mechanisms are key players in the phenotypic transition of tumor cells^14,15^. These phenotype changes that do not involve alterations in the DNA sequence can result in drastic changes not only in cell plasticity but also in drug resistance^16,17^. Besides, MAPK signaling pathways, which are deregulated in tumor^18,19^, have been constantly associated with chemoresistance in different type of cancer^20–22^. For instance, it has been reported that p38 and JNK MAPK pathways play a role in the control of the balance between autophagy and apoptosis in response to genotoxic stress^20^. Others have explored the role of ERK and p38 MAPK in breast cancer chemotherapy^21^. But, little is known concerning the involvement of MAPKs in tumor plasticity. Indeed, the molecular me chanisms that control cellular plasticity upon drug treatment remain to be fully established.

Here, using different breast cancer cell lines, we have evaluated the impact of drug stress on the immediate phenotype change by tracing the CSC marker CD24. We have shown that the rapid translocation of CD24 from cytosol to cell membrane was the triggering event for the acquisition of chemoresistance. In drug-resistant MDA-MB-231 cells, we have identified a tandem constituted by CD24 and p38 MAPK, where the continuous p38 activation controlled the overexpression of the survival marker Bcl-2. This phenotype enabled the cells to enter in slow-down of cell cycle, after which several weeks later, the dormant cells retrieved their capacity to proliferate. These cells were characterized by an increased resistance to drug and migratory capacity. Importantly, the use of p38 inhibitor was able to block the acquisition of drug resistance by impeding the upregulation of the anti-apoptotic protein Bcl-2. The correlation between translocation of CD24 and increased expression of Bcl-2 made in doxorubicin model was supported by the use of the TLR7 agonist Imiquimod that reduced cell proliferation without affecting phenotype switching nor Bcl-2 expression. Finally, we propose that the association of the p38 inhibitor, SB203580, with doxorubicin, a leading drug in clinic, could open up a new strategy in the fight against cancer.

## Results

### The translocation of CD24 from cytosol to membrane is an early event in breast tumor cells under drug stress

**Figure 1:**
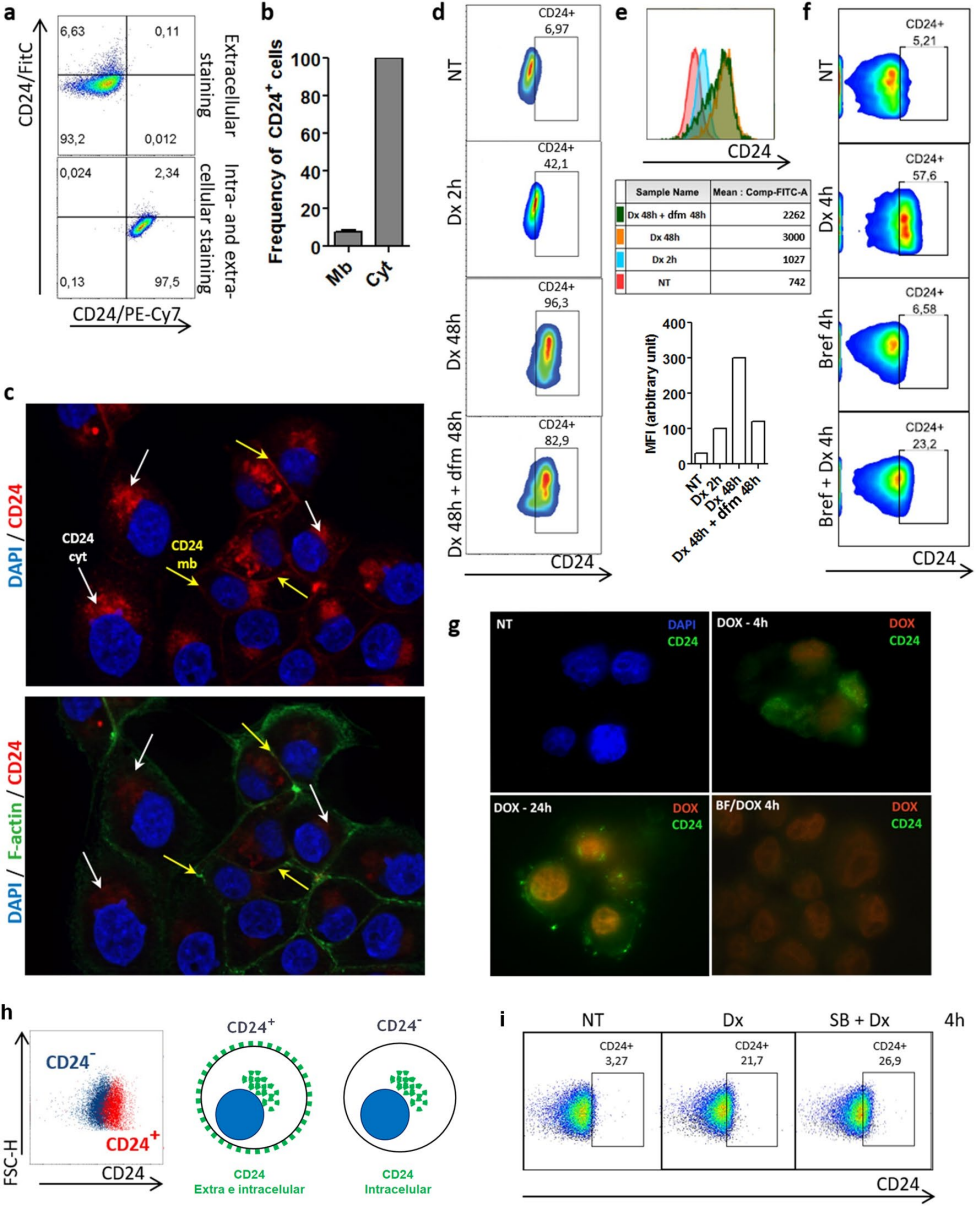
Translocation of CD24 from cytosol to cell membrane is an early event in MDA-MB-231 cells under drug stress. (**a**) Evaluation of the localization of CD24 in MDA-MB-231 cell line. MDA-MB-231 cells were stained with anti-CD24/FITC (extracellular staining) and with anti-CD24/PE-Cy7 (intracellular staining). Pseudocolor plots are representative of triplicates. (**b**) Frequency of CD24^+^ cells according to the detection of CD24 in membrane (mb) or in cytosol (cyt). The results are representative of four independent experiments (means ± SD of triplicates). (**c**) Visualization of CD24 localization by fluorescence microscopy. MDA-MB-231 cells were fixed, permeabilized and then stained using anti-CD24/PE-Cy7, anti-F-actin/Alexa488 and the nuclear dye DAPI. The yellow and white arrows indicate the mb CD24 and cyt CD24, respectively. (**d**) CD24^+^ cell enrichment in MDA-MB-231 population during doxorubicin (Dx) treatment. MDA-MB-231 cells were treated with Dx (0.6 μM) according to the indicated times followed or not by a drug-free medium (dfm) incubation. Then, an extracellular staining was performed using anti-CD24/FITC. The pseudocolor plots are representative of four independent experiments. (**e**) Histograms represent the shift of CD24 on x axis under different treatments. Table shows the MFI (median fluorescence intensity) values of surface CD24 expression calculated from figure d pseudocolor plots. Graph represents the means of triplicates obtained from the MFI calculation. (**f**) Reduction of CD24 translocation by brefeldin A in Dx-treated cells. MDA-MB-231 cells were pretreated or not with brefeldin A (1 μg/ml) 30 min before the addition of Dx (0.6 μM). Extracellular staining was performed using anti-CD24/PE-Cy7. The pseudocolor plots are representative of two independent experiments. (**g**) Effect of brefeldin A on CD24 localization in doxorubicin-treated cells visualized by fluorescence microscopy. MDA-MB-231 cells were treated with Brefeldin A prior to doxorubicin (0.6 μM). Then, cells were stained using anti-CD24/Alexa488 and DAPI without fixation and permeabilization. (**h**) Representative merged pseudoclor plots of CD24^+^ (red) and CD24^−^ (blue) subpopulations obtained from parental MDA-MB-231 cells after magnetic sorting. Schematic representation of CD24 localization in CD24^+^ and CD24^−^ suubpopulations. (**i**) Translocation of CD24 in CD24^**−**^ cells during doxorubicin treatment. CD24^−^ cells were treated with Dx (0.6 μM) for 4 h. Then cells were stained (extracellular staining) with anti-CD24/Pe-Cy7 antibody. The pseudocolor plots are representative of triplicates.

Phenotype switching, also commonly referred to as cell plasticity, is an important process observed during treatment of cancer, which was repeatedly associated with stemness. Here, using different breast cancer cell lines, we explored the dynamics of the CSC marker CD24 after doxorubicin treatment. At first, we sought to define the localization of CD24 in MDA-MB-231 cells by extra and intracellular staining. As shown by our flow cytometry results only about 5% of cells expressed CD24 in cell membrane (Fig. 1a,b). These finding corroborates other study^23^ and explains why MDA-MB-231 is considered CD24^low/−^. By contrast, a significant intracellular pool of CD24 was encountered in all the cells. Fluorescence microscopy confirmed the presence of extracellular (yellow arrows) and intracellular CD24 (white arrows) (Fig. 1c). After the treatment with doxorubicin at 0.6 μM—concentration representing the EC50 after 24 h of treatment calculated in MDA-MB-231 cell line—a cell phenotype switching occurred, which corresponded to an enrichment of the CD24^+^ subpopulation (Fig. 1d). Notably, MFI analysis showed an increase of surface CD24 density during drug treatment (Fig. 1e). This phenomenon occurred rapidly since ~ 42% of cells converted into CD24^+^ after 2 h to finally reach ~ 96% after 48 h of treatment, as visualized by flow cytometry (Fig. 1d). Importantly, the majority of cells remained positive even after a pause in the treatment (incubation in drug-free medium for 48 h after treatment) as visualized by the last pseudocolor plot in the Fig. 1d. The fact that this event was detected in the first hours of treatment excludes the possibility of a Darwinian selection of CD24^+^ cells. These results led us to hypothesize that the intracellular pool of CD24 immediately available might play a role in the CD24 translocation to cell surface. To support these data, MDA-MB-231 cells were sorted into CD24^+^ and CD24^−^ subpopulations (Fig. 1h) by using magnetic beads considering that CD24^+^ subpopulation expresses CD24 in both membrane and cytosol while CD24^−^ subpopulation lacks membrane CD24 expression as schematized in Fig. 1h. Then, CD24 localization was evaluated in CD24^−^ cells after doxorubicin treatment. As shown in the Fig. 1i, the translocation of CD24 occurred even in CD24^−^ population obtained after cell sorting since CD24^−^ cells were able to rapidly convert in CD24^+^ cells. Such data reinforce the idea that CD24^+^ cells enrichment during drug treatment does not correspond to a pre-selection of clones but to a drug-induced phenotype switching. In order to confirm this theory, we took the opportunity of using brefeldin A, an inhibitor of protein transport from endoplasmic reticulum to Golgi apparatus, to disturb the CD24 traffic after drug treatment. After flow cytometry analysis, we observed that when MDA-MB-231 cells were treated with brefeldin A prior to doxorubicin, the translocation of CD24 was reduced (Fig. 1f). These results were consistent with the fluorescence microscopy images obtained from doxorubicin-treated cells, which were stained with anti-CD24 without permeabilization to solely detect surface CD24 (Fig. 1g). To discard the hypothesis that the increased membrane CD24 expression could be partially due to an increased protein synthesis, we used actinomycin D, a DNA-transcription inhibitor. The incapability of actinomycin D to reduce the CD24^+^ phenotype enrichment in presence of doxorubicin indicated that the intracellular pool of CD24 was the main source of CD24 traffic in the presence of doxorubicin (data not shown). Importantly, using staurosporine, an inhibitor of protein kinase, a same phenomenon was observed resulting in the almost complete conversion of MDA-MB-231 cells into CD24^+^ cells after few hours (Supplementary Fig. 1).

**Figure 2:**
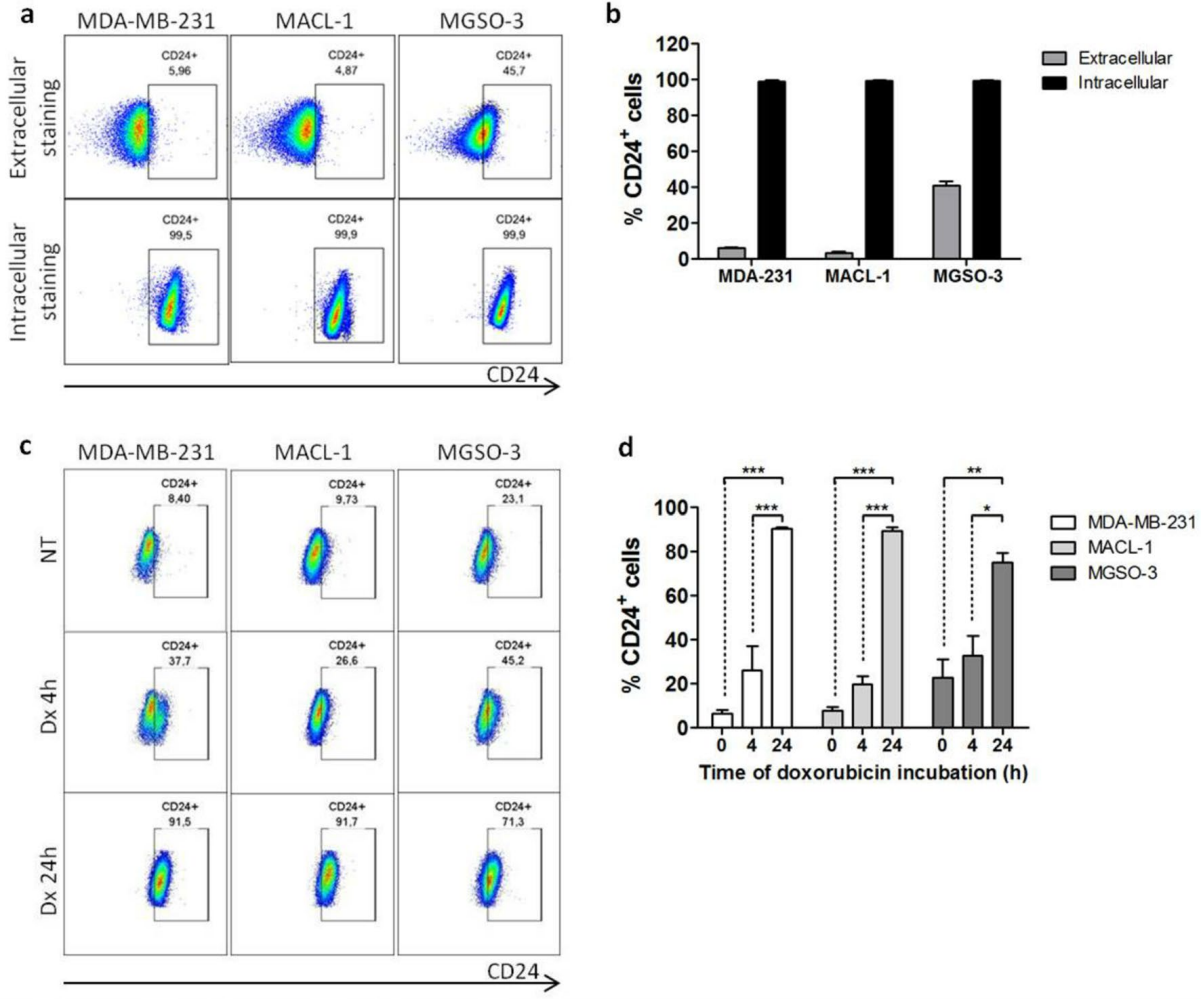
Translocation of CD24 from cytosol to membrane is observed in patient-derived breast cancer cells under drug stress. (**a**) Localization of CD24 in MDA-MB-231, MACL-1 and MGSO-3 cells after staining with anti-CD24/Pe-Cy7 (extracellular and intracellular staining). Dot-plots are representative of triplicates. (**b**) Frequency of CD24^+^ cells according to the detection of CD24 in membrane (extracellular) or in cytosol (intracellular). (**c**) CD24^+^ cell enrichment in breast cancer cell lines during Dx treatment. MDA-MB-231, MACL-1 and MGSO-3 cells were treated with Dx (0.6 μM) according to the indicated times and stained using anti-CD24/FITC. The dot-plots are representative of triplicates. (**d**) Frequency of CD24^+^ cells under Dx treatment according to the detection of CD24 in membrane. Data were plotted as means of triplicates ± SD. ***p < 0.001, **p < 0.01, *p < 0.05 (one-way ANOVA with bonferroni post-test).

Concerning MACL-1 and MGSO-3 breast cancer cell lines, obtained from Brazilian patients, the percentage of CD24^+^ cells was about 5% and 46% respectively, corroborating their classification by another study^24^. The presence of intracellular CD24 was also detected in the whole population of both cell lines, as observed in MDA-MB-231 population (Fig. 2a,b). In the same way, phenotype switching occurred in both cell lines which corresponded to an enrichment of the CD24^+^ subpopulation after 4 h doxorubicin treatment. The conversion rate in CD24^+^ cells reached ~ 90% for MDA-MB-231 and MACL-1 and ~ 70% for MGSO-3 cells (Fig. 2c,d) in cell lines treated for 24 h.

Therefore, we propose a new dynamic model of cell transition phenotype under drug stress which involves the translocation of intracellular CD24 that allows each breast cancer cell to convert into CD24^+^ cell.

### Translocation of CD24 in MDA-MB-231 cells treated with doxorubicin correlates with the upregulation of Bcl-2 expression and chemoresistance

**Figure 3:**
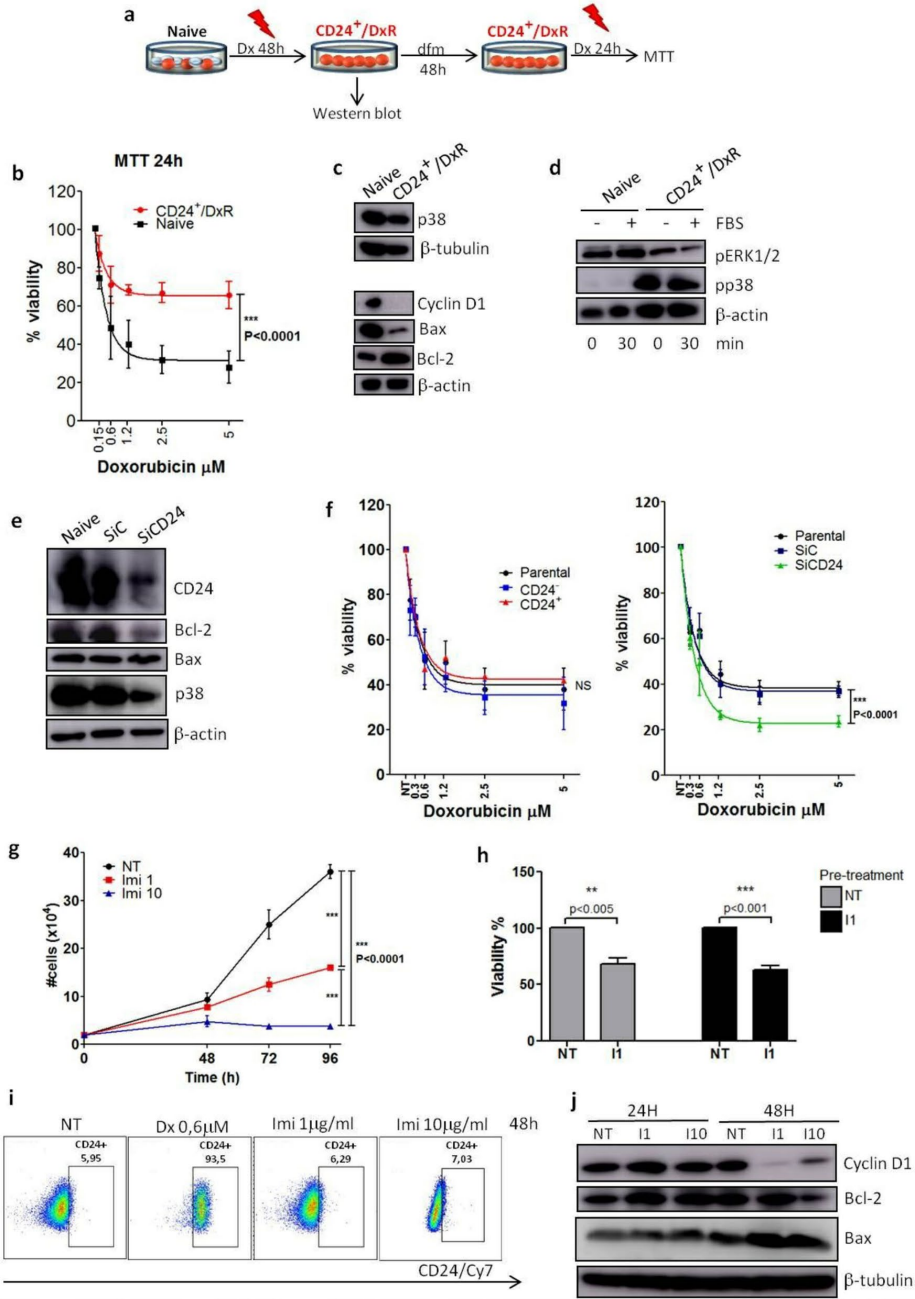
Translocation of CD24 is associated with chemoresistance and overexpression of Bcl-2. (**a**) Schematic shows experimental design used to obtain cell populations for MTT (**b**) and western blot assays (**c**,**d**). *dfm* drug-free medium, *Dx* doxorubicin, *CD24*^*+*^*/DxR* CD24^+^ doxorrubicin-resistant cells. (**b**) CD24^+^/DxR cells become tolerant to a second Dx treatment. Parental MDA-MB-231 cells were cultured in the presence of Dx at 0.6 μM for 48 h leading to the obtention of CD24^+^/DxR cells, which were cultured in a drug-free medium (dfm) for 48 h. CD24^+^/DxR and naïve MDA-MB-231 cells were submitted to Dx treatment for 24 h and cell viability was measured by MTT. The curves were fitted with non-linear regression as means of triplicates ± SD. ***p < 0.001 (two-way ANOVA with Bonferroni post-test). (**c**) CD24^+^/DxR cells are characterized by a reduction of cyclin D1 and Bax expression and increase of Bcl-2 level. Representative image of western blotting are shown and the data have been reproduced two times. (**d**) CD24^+^/DxR cells are characterized by a constitutive activation of p38 MAPK. Cells were starved for 2 h and then stimulated with serum (FBS) for 30 min. Cell lysates were immunoblotted with antibodies against the phosphorylated form of p38 (pp38) or ERK (pERK). (**e**) Downregulation of Bcl-2 and p38 expression in CD24 silenced (SiCD24) cells. SiC (control silenced) and SiCD24 (CD24 silenced) cell lysates were immunoblotted with the depicted antibodies. Representative images of western blotting are shown and the data have been reproduced three times. (**f**) Correlation between surface CD24 expression and sensitivity to Dx. Cell subpopulations were submitted to Dx treatment for 24 h and then cell viability was measured by MTT assay. Curves were fitted with non-linear regression as means of triplicates ± SD. ***p < 0.001 (two-way ANOVA with Bonferroni post-test). (**g**) Imiquimod reduces MDA-MB-231 cell proliferation. MDA-MB-231 cells were treated with Imiquimod. Then cells were counted and the curves were fitted as means of triplicates ± SD. ***p < 0.001 (two-way ANOVA with Bonferroni post-test). (**h**) Imiquimod does not induce chemoresistance to subsequent treatments on MDA-MB-231 cells. MDA-MB-231 cells were pre-treated with Imiquimod (1 μg/ml) for 96 h. After that, cells were incubated with a drug free medium for 48 h and then resubmitted to a second treatment with Imiquimod (1 μg/ml) for 96 h. Cell viability was evaluated by MTT. Data were plotted as means of triplicates ± SD. **p < 0.01 and ***p < 0.001 (T test). (**i**) Imiquimod does not induce CD24 translocation. MDA-MB-231 cells were treated with Dx or Imiquimod (1 μg/ml and 10 μg/ml) for 48 h. Then, an extracellular staining was performed using anti-CD24/Cy7. The pseudocolor plots are representative of two independent experiments. (**j**) Imiquimod reduces Bcl-2 expression. MDA-MB-231 cells were treated with Imiquimod (1 μg/ml or 10 μg/ml) in DMEM supplemented with fetal bovine serum (FBS) 10% for 24 h and 48 h. Cell lysates were immunoblotted with the depicted antibodies. The results are representative of two independent experiments.

Here, we investigated the molecular identity of the survival cells after 48 h of doxorubicin treatment, which we have named CD24^+^/DxR cells. The Fig. 3a represents the methodology used to obtain CD24^+^/DxR cells and schematizes the steps leading to MTT and western blot assays. At first, we tested the sensibility of the CD24^+^/DxR cells to respond to a second doxorubicin treatment using MTT assay. As shown in Fig. 3b, only a slight reduction of CD24^+^/DxR cell number was noted after a second treatment with doxorubicin. Meanwhile, the viability of naïve cells that received the treatment for the first time, declined below 40%. These data indicated that CD24^+^/DxR cells became tolerant. So, we sought to identify whether this phenotype change corresponded to a putative cell reprogramming in response to drug stress. By using western blot assay, we compared the protein profile of naïve and CD24^+^/DxR cells. When we focused on protein expression involved in cell proliferation or death, it was detected a significant increase of Bcl-2 expression, which was inversely correlated with Bax expression in CD24^+^/DxR (Fig. 3c). In addition, we observed a remarkable decrease of cyclin D1, a regulator of cell cycle progression, suggesting that acquisition of drug tolerance controlled by Bcl-2 expression may also require cells to exit the cell cycle (Fig. 3c). In previous studies, we have focused on the role of p38 MAPK and ERK1/2 in the proliferation of MDA-MB-231 cells^25,26^, so we analyzed their activation profile in the resistant CD24^+^/DxR cells. Interestingly, the phenotype switching was accompanied by a strong and continuous activation of p38 MAPK at the detriment of ERK1/2 MAPK (Fig. 3d). Then, we verified the relationship between CD24 and Bcl-2 by silencing CD24 using interference-RNA. In CD24-silenced cells (SiCD24), a decreased Bcl-2 and p38 expression was observed (Fig. 3e). This may explain the reduced capacity of SiCD24 cells to resist to drug treatment in all the doxorubicin concentrations tested when compared to control-silenced (SiC) and parental MDA-MB-231 cells (Fig. 3f). Interestingly, CD24^−^ subpopulation, obtained from magnetic sorting, presented a drug sensibility similar to CD24^+^ subpopulation and parental MDA-MB-231 cells. This is in accord with the fact that even CD24^−^ converted into CD24^+^ after drug treatment (Fig. 1i).

Next, we sought to treat MDA-MB-231 cells with a drug capable to reduce cell proliferation without inducing immediate death. We tested the efficacy of the TLR7 agonist Imiquimod, previously used in skin cancer cutaneous metastatic breast cancer treatment^27–29^, in reducing MDA-MB-231 cell proliferation. At the concentration of 1 μM, a significant decrease of cell proliferation was observed while a total blocking of cell replication was noted at 10 μM during the period of experiment (Fig. 3g). No significant cell death was observed in the first 48 h in the presence of both concentrations of Imiquimod. In such context, we tested the capacity of cells to respond to a second treatment 96 h after the first dose of Imiquimod. As shown in Fig. 3h, a similar pattern of cell viability was observed when a unique dose or two subsequent treatments with the TLR7 agonist were used indicating the absence of chemoresistance. To correlate phenotype switching and chemoresistance, we traced the CD24 marker in Imiquimod treated cells. We confirmed that no enrichment of CD24^+^ cells occurred during the treatment (Fig. 3i). In accordance with this, no upregulation of Bcl-2 expression was detected in cells treated with Imiquimod (Fig. 3j). These data reemphasize a link between CD24 translocation, upregulation of Bcl-2 expression and chemoresistance.

### Association between CD24 translocation and activation of p38 in the chemoresistance acquisition phenotype of MDA-MB-231 breast cancer cells

According to the Fig. 3d, p38 phosphorylation was stronger, constitutive and independent on serum in CD24^+^/DxR cells which contrasts with the serum-dependent activation of p38 in MDA-MB-231 cells under proliferative conditions. This suggests that p38 activation in diverse configurations can cause different outputs. This led us to investigate its role in the phenotype switching of MDA-MB-231 cells in the presence of doxorubicin.

**Figure 4:**
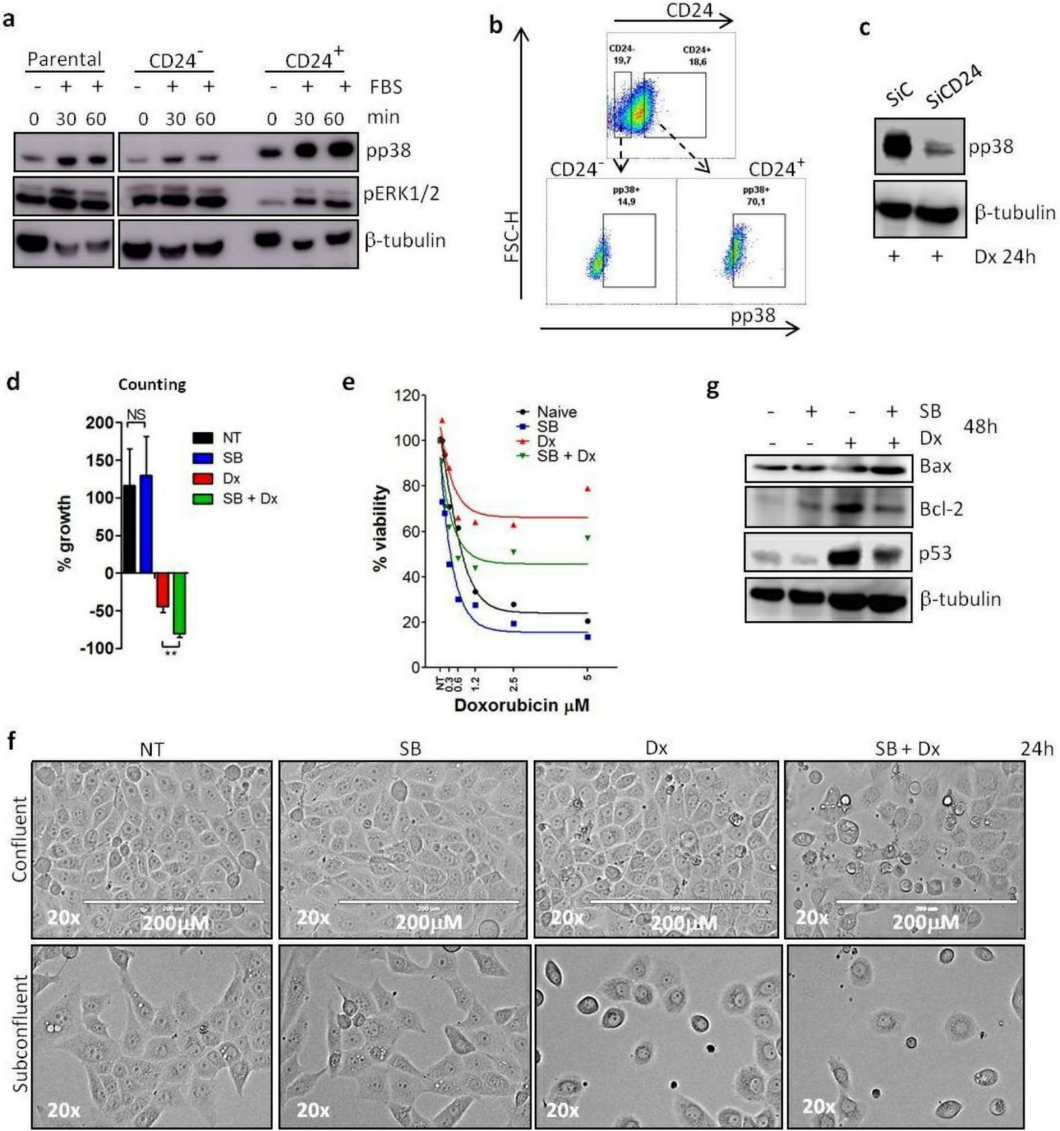
Inhibition of p38 activity impedes phenotype switching of MDA-MB-231 cells under doxorubicin treatment. (**a**) Differential activation of MAPKs in CD24^+^ and CD24^−^ subpopulation. Parental and the magnetic sorted CD24^+^ and CD24^−^ cells were starved for 2 h. Then, cells were submitted to time kinetic using fetal bovine serum (FBS) as stimulus. Cell lysates were immunoblotted with the depicted antibodies. The results are representative of two independent experiments. (**b**) Higher p38 phosphorylation (pp38) MAPK in CD24^+^ cells. Parental MDA-MB-231 cells were stained with anti-CD24/Pe-Cy7 (extracellular staining) and anti-pp38/FITC (intracellular staining). The pseudocolor plots are representative of triplicates. (**c**) Decreased phosphorylation of p38 (pp38) in SiCD24 cells treated with Dx. SiC and SiCD24 cells were treated with Dx (0.6 μM) for 24 h. Cell lysates were immunoblotted with the depicted antibodies. All blots were performed at least twice. (**d**) Increased efficiency of Dx when associated with the p38 inhibitor SB203580. Cells were treated with SB203580 (SB) at 10 μM or/and Dx at 0.6 μM for 48 h. Then, cells were counted and the growth percentage was calculated based on the initial number of cells before each treatment (% growth = [fn/in − 1] × 100)^#^. Countings were performed in three independent experiments. (**e**) Reduction in the acquisition of Dx resistance in the presence of the p38 inhibitor SB203580. MDA-MB-231 cells were pre-treated with SB203580 (10 μM) and/or Dx (0.6 μM) during 48 h before the addition of Dx at different concentrations for 24 h and then cell viability was measured by MTT assay. Curves were fitted with non-linear regression as means of triplicates. (**f**) Representative light microscopy images of cell population at confluence (10^6^ cells) or subconfluence (5 × 10^5^ cells) density treated with SB203580 (10 μM) and/or Dx (0.6 μM) for 24 h. Cell density and morphology were evaluated by microscopy (Evos Skedda). The microscopy images are representative of triplicates. (**g**) Increased expression of Bax but decreased expression of Bcl2 and p53 after cell treatment with the association of SB203580 and Dx. MDA-MB-231 cells were treated with SB203580 (10 μM) and/or Dx (0.6 μM) and cells were lyzed after 48 h treatment. Representative western blotting is shown and the data have been reproduced twice. ^#^*fn* final number of cells, *in* initial number of cells.

So, our next question was whether there was a privileged relationship between CD24 and p38. At first, we explored this in MDA-MB-231 cells under growing culture conditions. After magnetic sorting, we evaluated the status of MAPK activation with western blotting after cell stimulation with serum according to the kinetic presented in the Fig. 4a. As clearly shown, CD24^+^ cells phosphorylated p38 in a more pronounced way than CD24^−^ subpopulation. In contrast, a higher phosphorylation of ERK1/2 was observed in the CD24^−^ and parental MDA-MB-231 cells. The results obtained by flow cytometry confirmed the correlation between surface CD24 expression and preferential p38 phosphorylation. About 70% of the CD24^+^ cells phosphorylated p38, while the activation of this MAPK was observed in only ~ 15% of the CD24^−^ cells (Fig. 4b).

Another piece of evidence that demonstrates a link between CD24 and p38 is presented in the Fig. 4c. According to the western blotting, siRNA-mediated knockdown of CD24 decreased the phosphorylation of p38 when cells were submitted to doxorubicin treatment, which indicated that the absence of CD24 jeopardized the cell capacity to induce activation of p38 MAPK.

The sustained p38 activation in CD24^+^/DxR cells makes it a prime target. In this context, we used SB203580, a p38 activity inhibitor^30^ to evaluate its impact on drug resistance. According to the results obtained by cell counting, the combination of SB203580 and doxorubicin was more efficient in reducing cell number than doxorubicin alone. Importantly, SB203580 alone was unable to impact on cell viability (Fig. 4d). Concerning the results observed by MTT assay, the inhibition of p38 was benefit from two aspects: first, SB203580 sensitized MDA-MB-231 cells to the therefore doxorubicin treatment (blue line vs black line). Second, SB203580 disrupted the resistant-phenotype acquired by the cells that received two consecutives doxorubicin treatments (green line vs red line) (Fig. 4e). The effect of the drug association may be considered as synergistic since the total effect of combined SB203580 and doxorubicin was greater than the sum of the individual effects of each drug.

To visualize these results, we performed the capture of light microscopy images of cells treated with the drug pair. These experiments were performed under sub-confluence or confluence conditions to exclude the influence of fluctuating environment. A direct impact of doxorubicin on MDA-MB-231 cells was observed after 24 h of treatment marked by a decreased cell number and changes in morphology. The association of SB203580 and doxorubicin exacerbated the cell phenotype changes under confluent and sub-confluent conditions. The results confirmed that the efficiency of the drug pair constituted by doxorubicin and SB203580 was superior in killing cells in both plating conditions (Fig. 4f).

Consistent with the above results, western blots showed that SB203580 prevented the increase in Bcl-2 expression induced by doxorubicin (Fig. 4g). Further, in MDA-MB-231, which has high levels of a mutant p53, it has been described that mutant p53 can contribute to the suppression of apoptosis^31^. In line with this, SB203580 was also able to reduce the expression of p53 in doxorubicin treated cells (Fig. 4g).

Taken together, these results suggest that targeting p38 can overcome adaptive resistance to doxorubicin treatment.

### CD24/DxR cells become proliferative after a long-lasting period in dormancy

The capacity of slow-cycling cells to reentry into cell cycle has been a topical debate for quite some time. As reported above, CD24^+^/DxR cells have adopted a slow-down cell cycle after doxorubicin treatment. This was evidenced by a reduction of cyclin D1 (Fig. 3c).

**Figure 5:**
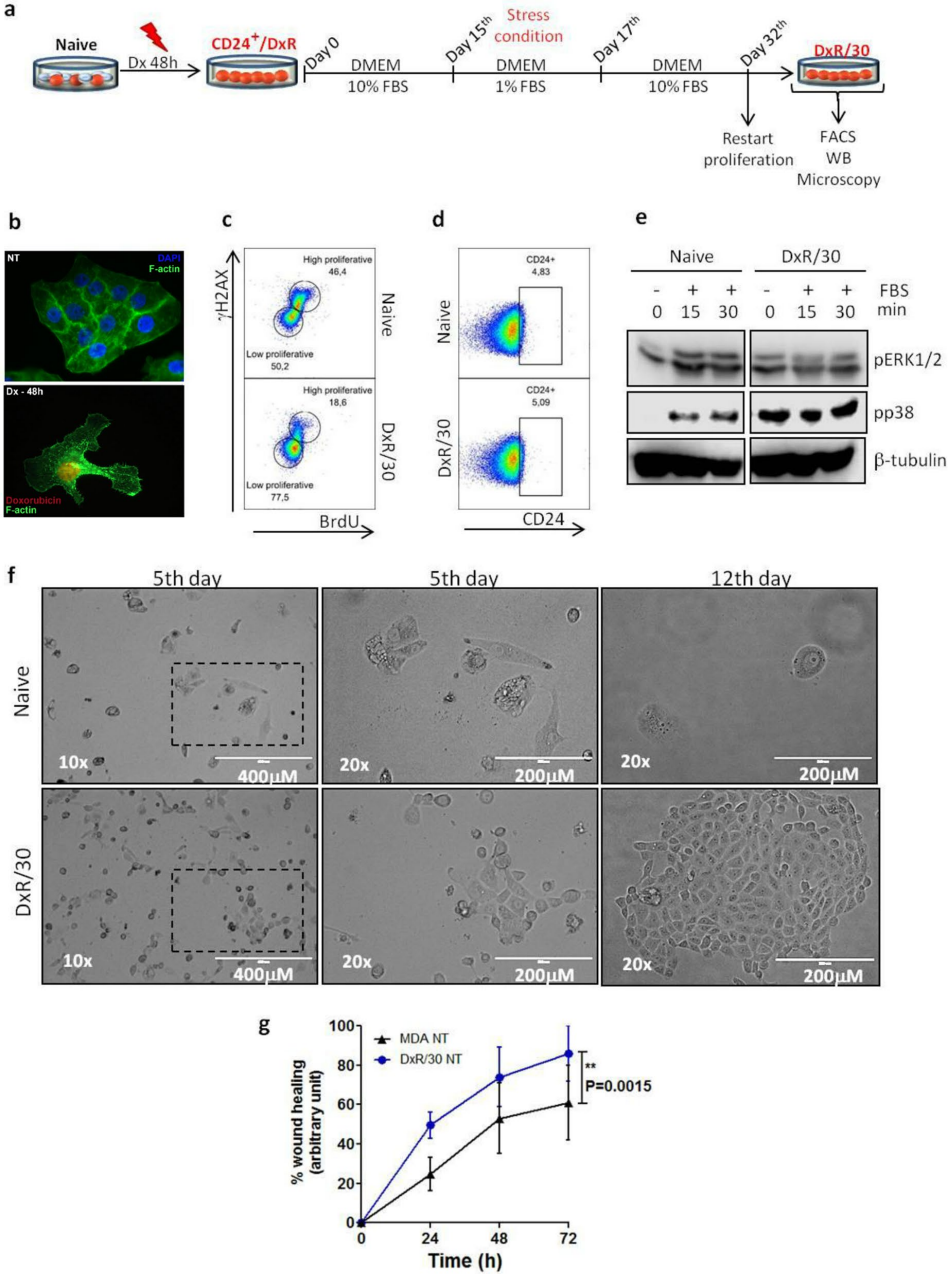
CD24^+^/DxR cells become proliferative and more invasive than naïve cells after a long-lasting period in drug-free environment. (**a**) Schematic shows the journey of CD24^+^/DxR cells from their switching to CD24^+^ phenotype to their entry in slow- cycling state and reversion into proliferative cells. CD24^+^/DxR cells were exposed to a stress condition (fetal bovine serum starvation = 1% FBS) during 48 h. (**b**) Differences in morphology between naïve MDA-MB-231 and CD24^+^/DxR cells. Naïve MDA-MB-231 and CD24^+^/DxR cells were fixed, permeabilized and stained using anti-F-actin/Alexa488 and the nuclear dye DAPI. (**c**) CD24^+^/DxR cells retrieve their capacity to proliferate (named as DxR/30 or revertant). Naive MDA-MB-231 and DxR/30 cells were incubated with 10 μM of BrdU (thymine analogue base) for 4 h. Then, cells were stained with anti-BrdU/PerCP-Cy5.5 and anti-γH2AX/Alexa647 (intracellular staining). The low and high proliferative subpopulations were defined and analyzed. The pseudocolor plots are representative of triplicates. (**d**) DxR/30 cell population returns to the basal ratio of CD24^+^ cells. Cells were stained with anti-CD24/Pe-Cy7 (extracellular staining). Pseudocolor plots are representative of triplicates. (**e**) Constitutive activation of p38 MAPK in DxR/30 cells. After starvation for 2 h, cells were stimulated with serum in different times. Cell lysates were immunoblotted with the depicted antibodies. All blots were performed at least twice. (**f**) Higher resistance of DxR/30 cells to Dx treatment. MDA-MB-231 and DxR/30 cells were treated with Dx (0.6 μM) for 24 h. Then cells were counted, re-seeded in the same density and cultured in normal conditions (DMEM 10% FBS). Cell density and morphology were analyzed at 5 and 12 days post-Dx treatment. (**g**) DxR/30 cells are more invasive than MDA-MB-231 cells. MDA-MB-231 and DxR/30 cells were seeded (8 × 10^5^) in 6 wells plate and a scratch performed according to Mat and Med. Closing-time percentage was calculated based on the initial scratch size of each cell type. Curves were fitted as means of duplicates ± SD. **p < 0.01 (two-way ANOVA with Bonferroni post-test).

So, we sought to monitor CD24^+^/DxR cells to evaluate the reversibility of their dormant state according to the scenario presented in Fig. 5a. As shown in Fig. 5b by fluorescence microscopy, CD24^+^/DxR cells have acquired an enlarged cell morphology, which is a hallmark of dormant cells (Fig. 5b). CD24^+^/DxR cells were cultured in drug-free medium for a long period, and then, submitted to a serum deprivation for 2 days followed by culture in medium with 10% of serum. In such conditions, we observed the emergence of revertant cells (named DxR/30) which reacquired the ability to proliferate as confirmed by their capacity to incorporate BrdU (Fig. 5c).

The percentage of CD24^+^ cells in the revertant-population recovered the levels observed in naïve MDA-MB-231 cell population (Fig. 5d). Importantly, even in the absence of the drug, the phosphorylation of p38 in DxR/30 remained strong and constitutive, which indicates that DxR/30 cells have conserved some features of their precedent states while eliminated others (Fig. 5e).

When we evaluated the drug resistance of DxR/30 cells, more than 1 month after the first treatment, the cells remained tolerant to doxorubicin. As shown in the light microscopy images, the morphology of these cells appeared little affected after treatment when compared to naïve MDA-MB-231 treated cells. More surprisingly, DxR/30 cells retained their capacity to proliferate even in the presence of drug (5 days). After 12 days of treatment, DxR/30 cells have re-colonized the plastic dishes (Fig. 5f). One of the hypothesis is that DxR/30 cells might have a competitive advantage over naïve cells under drug stress due to their constitutive phosphorylation of p38. Finally, we evaluated the migratory capacity of these cells by using the in vitro wound healing assay, which is based on the creation of an artificial gap, so called “scratch”, on a confluent cell monolayer. Images were captured every 24 h during cell migration, the scratch was measured and a comparison of time required to close the scratch between naïve cells and DxR/30 was performed. The incubation time was determined at 48 h when the faster moving cells DxR/30 were just about to close the scratch. The confirmation of the migratory capacity of both cells was made with the Image J software (Fig. 5g). These data confirm that DxR/30 cells have acquired a new identity, conserving some traces of naïve cells and acquiring markers of chemoresistance.

According to our data, slow-cycling cells under stress may reentry into cell cycle, which gave to cells new properties, including higher drug resistance and higher migratory capacity, reaffirming that they have acquired a new identity.

## Discussion

Solving the drug resistance problem in cancer remains a big challenge, which includes the need for a better tracking of “persistent” tumor cells after treatment. Robust evidence showed that the CSC subpopulation is enriched after chemotherapy, suggesting that this subset is responsible for the majority of treatment failure^32,33^. In this context, the relevant question is how can we summarize the tumor heterogeneity using a few pertinent markers to predict cell behavior under drug treatment. Here, using CD24 as marker, we provide new insight about the mechanisms leading to the emergence of “persistent” subpopulation during chemotherapy. By monitoring the CSC marker CD24 during doxorubicin treatment in MDA-MB-231 breast cancer cells, we have shown the uniform conversion of CD24^−^ population into CD24^+^ cells, which we have identified as drug-resistant cells.

However, the translocation of CD24 observed in MDA-MB-231 cells under drug stress is not restricted to doxorubicin treatment since the cell incubation with staurosporine, another anticancer drug, induced a similar cell phenotype switching. Further, when we treated other breast cancer cell lines like MGSO-3 and MACL-1 with doxorubicin an increased expression of CD24 at cell surface was also detected. Importantly, MDA-MB-321, MGSO-3 and MACL-1 cell lines share some important features like metastatic properties, aggressive character, the presence of an intracellular pool of CD24 and the capacity to modulate membrane CD24 expression under stress. According to our data, CD24 translocation could represent a more global phenomenon linked to tumor cell plasticity that may overtake specific mechanism of resistance. These different considerations highlight the relevance of the use of CD24 as a marker of cell plasticity and transition state in aggressive breast tumor cells.

In the same line, Goldman et al. have reported that the treatment of breast or ovarian cancer cells with high concentration of taxanes results in the generation of “persistent” cells, which are defined by a transition towards a CD44^Hi^CD24^Hi^ expression status^12^. Others studies have demonstrated an enrichment for CSC-like phenotype after chemotherapy in glioblastoma^34^ indicating how the phenomenon is ubiquitous.

Here, we provide evidence that translocation is the most reasonable hypothesis to explain a rapid appearance of CD24 (within 2 h) at the cell surface under drug stress**.** According to that, each given breast tumor cell may convert into CD24^+^ phenotype made possible by the presence of an intracellular pool of CD24. Such findings suggest that the presence of CSC marker in cell membrane does not correspond to a clonal identity but rather to a state as also claimed by Dirske et al.^34^. The systematic conversion of CD24^−^ into CD24^+^ after chemotherapy demonstrates that cell plasticity emerges as an important contributor to therapy escape. This was verified when we used the TLR7 agonist Imiquimod, capable of reducing MDA-MB-231 cell proliferation without inducing translocation of CD24^+^, nor changes in the expression of the anti-apoptotic protein Bcl-2. We were able to correlate these data with the absence of resistance to a second Imiquimod treatment. In this regard, great efforts have been directed towards finding small molecules to inhibit these anti-apoptotic Bcl-2 family proteins, and thus, to tackle anti-apoptotic adaptation of tumor cells^35^.

Hence, targeting cell plasticity should provide a unique opportunity to improve the efficiency of existing therapies. Tumor cells have been shown to hijack signaling pathways involved in reprogramming to become plastic and evolve towards drug-refractory cells. So, the identification of these signaling pathways may be a key to hamper chemoresistance. The tandem constituted by CD24 and the p38 MAPK appears crucial in the cell fate of MDA-MB-231 population. A preferential use of p38 in CD24^+^ cells have been noted under normal conditions, which has been amplified under drug treatment in accord with the dynamics of CD24. The relationship between CD24 and p38 is supported by the incapability of SiCD24 cells to activate p38 under drug stress. Indeed, we hypothesized that the change in the dynamics of p38 activation, which turned sustained early after CD24 translocation, is linked to the increased surface CD24 expression observed in doxorubicin treated cells. In this regard, a very recent paper reported that MAPK cascade signaling dynamics (transient to sustained activation) may be controlled by the activation kinetics of a given membrane receptor and not necessarily by the intracellular topology of the kinase networks. In the same study, it was proposed that redirecting signal dynamics may be a more fruitful and effective approach than controlling receptor activation in pathologic situation^36^.

In other tumor models, doxorubicin resistance mechanism seems to use some common molecular features involving p38 signaling pathway. Downregulation of stemness/EMT pathways like Notch-1 and Wnt/β-catenin, alongside of downregulation of STAT3, a well-known p38 downstream target, avoided doxorubicin-resistance in enriched CD44^−^/CD24^+^ subpopulation of MCF-7^37^. Pharmacological downregulation of AKT, another p38 downstream target, also reduced doxorubicin resistance in non-small cell lung cancer^38^. Yet, downregulation of STAT3 and Bcl-2 by miRNA reduced doxorubicin-resistance in breast cancer^39,40^. This is why we considered the profile of p38 activation as an important marker of the resistant cell identity (CD24^+^/DxR) and a key target to hinder phenotype switching during doxorubicin treatment.

The relevance of this strategy was verified when we compared the protein expression pattern of CD24^+^/DxR cells and naïve MDA-MB-231 cells when they were treated with doxorubicin in the presence of the p38 inhibitor SB203580. The overexpression of Bcl-2, indicator of adaptative cellular reprogramming and also marker of premature senescence^41–43^ observed in CD24^+^/DxR was not detected in cells treated with the combination of SB203580 and doxorubicin. The benefit of this association was translated into a synergism of the cytotoxic effect of the drug pair. The impact of SB203580 on cell fate may not only correlate with Bcl-2 expression but also with p53 levels since we have observed a modulation of p53 expression in the SB203580 treated cells. Recently, p53 was also considered as a marker of cell reprogramming and acquisition of stemness^44^. These data sustain the idea that p38 is an active player in drug resistance by inducing cell identity changes, notably increasing cell survival marker expression.

To better assess the long term consequences of the rapid phenotype switching after drug treatment we have evaluated whether the slow-cycling state of CD24^+^/DxR cells was reversible after several weeks in drug-free medium since several studies have demonstrated the reversibility of senescence^45–48^. In fact, the revertant cells (named DxR/30), recovered their capacity to proliferate and importantly, have gained higher drug resistance and stronger migratory properties. This could be attributed to the fact that p38 is constitutively activated in these cells. Interestingly, in the absence of the drug pressure and in proliferative conditions, the DxR/30 population retrieved a basal level of CD24^+^ cells. This follows mathematical models that tend to establish that tumor cell populations always maintain its heterogeneity at fixed ratio in dynamic conditions like proliferation^49^.

Thus, here we have established a model to accompany the journey of MDA-MB-231 cells after drug treatment starting from their switching to CD24^+^ phenotype to their entry into slow-cycling state and reversion into proliferative cells. Notably, the sustained p38 phosphorylation observed in all the post-treatment stages may also indicate the participation of this MAPK in the network that successfully promotes the formation of metastasis at distant organs. Previous studies have shown that the EMT (Epithelial Mesenchymal Transition) program has been involved in the distant metastases frequently detected following chemotherapy^50^ which could also include p38. The monitoring of cell evolution throughout this journey has shown that they have conserved some features of previous state while eliminated others. In other words, they have acquired hybrid properties corresponding to a new identity.

**Figure 6:**
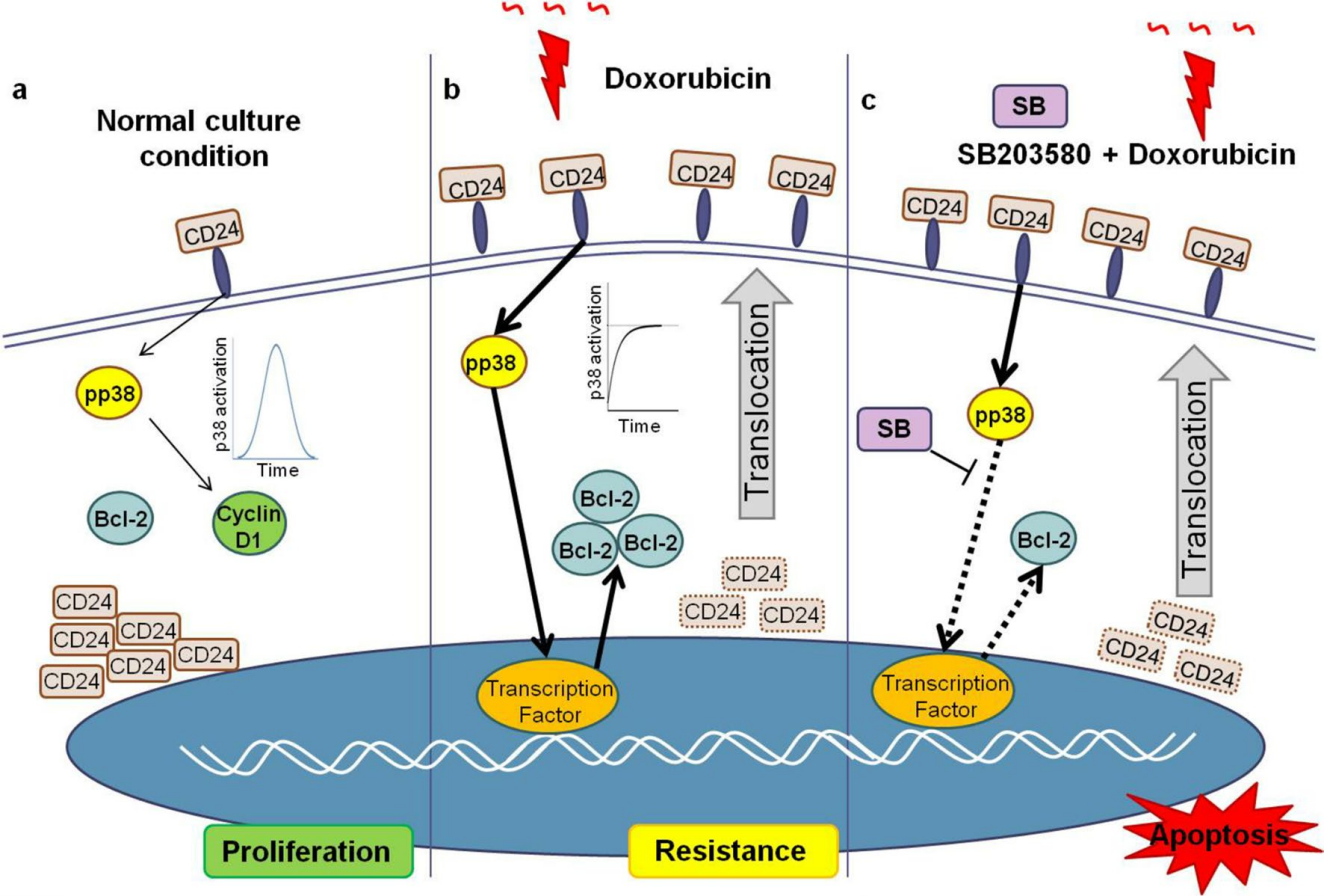
Schematic shows breast cancer cell population phenotype changes under drug stress toward a drug tolerant state arising through translocation of CD24 and sustained p38 activation. (**a**) In proliferative conditions, CD24 is mainly detected in the cytosol of all cells and the population is characterized by a low frequency of CD24^+^ cells (cells carrying CD24 in membrane). The transient phosphorylation of p38 leads to cyclin D1 expression and cell cycle progression. (**b**) Under drug treatment, CD24 translocation associated with an increased CD24 density on cell membrane occurs in every given cell. In accord with this, a sustained and strong p38 activation, an increased Bcl-2 expression are observed indicating a phenotype switching. (**c**) This transition state is sensitive to p38 inhibition since the use of a specific inhibitor, like SB203580, in association with doxorubicin reduces Bcl-2 expression leading to an increased cell death.

This study reaffirms how a better understanding of the biology and molecular drivers of the cellplasticity will enable identification of new anticancer targets. The pertinence of using a leading drug with an inhibitor of cell reprogramming was illustrated by the high efficiency of the combination of p38 inhibitor and doxorubicin on the killing of aggressive MDA-MB-231 breast cancer cell line as schematized in the Fig. 6.

## Materials and methods

### Reagents

*Thiazolyl Blue Tetrazolium Blue *(MTT), Doxorubicin, Staurosporine and RIPA buffer were purchased from Sigma-Aldrich. p38 inhibitor SB203580 and Imiquimod were obtained through InvivoGen. Protease cocktail inhibitor and phosphatase cocktail inhibitor (Roche) were acquired from Sigma Aldrich. Monoclonal antibodies against phospho-p44/42 (ERK1/2), phospho-p38 MAPK, p38 MAPK, cyclin D1, β-tubulin, β-actin and peroxidase-conjugated goat anti-mouse IgG and goat anti-rabbit IgG were obtained from Cell Signaling Technology and anti-Bcl-2 (Novex) and anti-Bax (Invitrogen) from Thermo Fisher Scientific. Apoptosis, DNA Damage and Cell Proliferation Kit from BD Biosciences and *Lipofectamin 300 transfection reagent* (Invitrogen) from Thermo Fisher Scientific. SiRNAs for CD24 silencing (Cat# Hs01 00232148) *and for control (Cat# SIC001)* were synthetized by Sigma-Aldrich. *PVDF membranes (0.45 μM) and Luminat (Millipore) were acquired from Merck and nitrocellulose membrane (022 μM) from Bio-Rad.*

### Cell culture conditions

MACL-1 and MGSO-3 cell lines were previously established by Goes et *al*. from Brazilian patients with breast cancer^51^. MDA-MB-231 cell line was obtained from ATCC (USA). Cells were grown at 37 °C in a humidified atmosphere of 5% CO_2_ in DMEM supplemented with 10% heat inactivated fetal bovine serum (FBS). For starvation conditions, cells were incubated with serum-free DMEM for 2 h before the addition of the respective stimuli.

### Obtaining of the resistant cell clones from parental MDA-MB-231

The procedures to obtain MDA-MDA-231 cells resistant to doxorubicin (named CD24^+^/DxR cells) are schematized in Fig. 4a. Briefly, MDA-MB-231 cells were incubated with doxorubicin at 0.6 μM for 48 h. The remaining cells, named CD24^+^/DxR, were trypsinized and used in the respective experiments. These cells were characterized as non-proliferative. DxR/30 achievement is schematized in Fig. 5a. After obtaining CD24^+^/DxR cells, these cells were cultured with DMEM 10% of fetal bovine serum for 15 days, then cells were cultured under stress condition (DMEM 1% of fetal bovine serum for 48 h) following by incubation in normal culture conditions until they recover their proliferative activity. From then, cells were named DxR/30. In order to obtain MDA-MDA-231 cells resistant to Imiquimod, MDA-MB-231 cells were incubated with Imiquimod at 1 μg/ml for 96 h. The remaining cells were trypsinized and used in the respective experiments.

### MTT (viability assay)

For doxorubicin assay, MDA-MB-231 cells (10^4^/well) were plated in 96-well plates in DMEM with 10% serum. Cells were incubated for 24 h with different stimuli according to the figures. For Imiquimod assay, MDA-MB-231 cells (10^4^/well) were plated in 24-well plates in DMEM with 10% serum. Cells were incubated for 96 h with different stimuli according to the figures. After the respective treatment time, the medium was removed and cells were incubated with 50 μl/well of DMEM 10% FBS plus of 40 μl/well of MTT (5 μg/ml) for doxorubicin assay or μl/well of DMEM 10% FBS plus of 40 μl/well of MTT (5 μg/ml) for Imiquimod assay for 2 h at 37 °C. After removing the medium, 50 μl (for doxorubicin) or 200 μl (for Imiquimod) of DMSO were added to dissolve the crystals of formazan and the absorbance was measured in a microplate reader at a wavelength of 595 nm. A value of 100% was assigned to untreated control cultures. Results were derived from at least three independent sets of triplicate experiments.

### RNA silencing

MDA-MB-231 cells were seeded in 24-well plates at a density of 2 × 10^4^ cells/well. After 24 h of incubation, the cells were transiently transfected with short interfering RNA (siRNA) specific for CD24 or control siRNA at a final concentration of 100 nM by using Lipofectamin 3000 Transfection Reagent according to manufacturer’s instructions. Briefly, siRNA and Lipofectamin were diluted separately in serum-free OPTI MEM. Then, the diluted Lipofectamin and siRNA were mixed (1:1 v/v). After 5 min, the mix was added drop-wise onto the cells under their normal growth conditions and after 48 h silenced cells were used in the different assays. CD24 silencing was confirmed by western blotting.

### Western blotting

For protein expression, cells were harvested, counted and lysed in RIPA buffer supplemented with phosphatase and protease inhibitor cocktail according to the manufacturer’s instructions. For the evaluation of the phosphorylated form of MAPKs, cells were seeded in 6-well plates (10^6^ cells/well) and were starved for 2 h in free-serum medium (SFM). Then, cells were treated for 30–60 min with medium containing 10% FBS as stimuli. At the end of treatment cells were lysed in RIPA buffer supplemented with phosphatase and protease inhibitor cocktail according to the manufacturer’s instructions. Protein lysates were separated by polyacrylamide gel electrophoresis on 10% or on 15% gels when concerned histone extract, and electrotransferred to PVDF-0.45 μM membranes (Milipore) or nitrocellulose-0.22 μM (BioRad). Membranes were blocked overnight with 5% dry milk and were incubated with primary antibodies in 5% BSA also overnight. After incubation with the peroxidase-conjugated secondary antibody for 1 h, protein expression was detected using Luminat HRP reagent (Milipore) and analyzed using LAS-4000 imaging system (Fuji) or C-DiGit Blot Scanner (Li-Cor).

### Magnetic sorting

The different cell subpopulations (CD24^+^ and CD24^−^) were sorted from parental MDA-MB-231 cells using *CD24 MicroBead Kit* (*MACS Miltenyi Biotec*) following manufacturer’s instructions. Briefly, the CD24^+^ cells were indirectly magnetically labeled with CD24-Biotin antibodies and Anti-Biotin MicroBeads. Then the cell suspension was loaded onto a MACS Column, which is placed in the magnetic field of a MACS Separator. The magnetically labeled CD24^+^ cells were retained within the column. The unlabeled cells (CD24^−^ cells) ran through; this cell fraction was thus depleted of CD24^+^ cells. After removing the column from the magnetic field, the magnetically retained CD24^+^ cells can be eluted as the positively selected cell fraction. The purity of the sorted populations was verified by Flow Cytometry.

### Flow cytometry analysis

To evaluate the proliferative activity of cells, an assay using the Apoptosis, DNA Damage, and Cell Proliferation Kit from BD Biosciences was realized in accord with the manufacturer’s instructions. Cells were cultured as indicated in the figure legends and washed/blocked in staining buffer (PBS 4% fetal bovine serum—v/v). Cells were fixed and permeabilized using *CitoFix/CitoPerm* reagent for 20 min on ice, nucleus was permeabilized using *CitoFix/CitoPerm* Plus for 10 min on ice and finally treated with DNAse (30 μg/10^6^cells) for 1 h at 37 °C in humidified chamber. Then, cells were simultaneously stained with anti-BrdU/PerCP-Cy5.5 and anti-γH2AX/Alexa647 (BD Biosciences) fluorescent antibodies for 20 min at room temperature. Between every step cells were washed with 1 × PBS/PermWash (BD Biosciences). Concerning CD24 and p38 staining**,** the following step by step was realized**.** For extracellular staining, MDA-MB-231 cells were washed/blocked in staining buffer (PBS 4% fetal bovine serum—v/v) and were labeled using anti-CD24/Pe-Cy7 (eBioscience) or anti-CD24 (BD Pharmigen) for 20 min on ice, and when necessary, followed by FITC-conjugated equivalent secondary antibody (BD Pharmigen) for more 20 min on ice. For intracellular staining, after extracellular labeling, cells were fixed and permeabilized using *CitoFix/CitoPerm* reagent for 20 min on ice. Then, cells were incubated with anti-CD24/Pe-Cy7 (eBioscience) and/or with anti-pp38 (Cell Signaling) for 20 min at room temperature, and when necessary, followed by FITC-conjugated secondary antibody (BD Pharmigen) for 20 min at room temperature. Between every step cells were washed with 1 × PBS/PermWash (BD Biosciences). After labeling protocols, cells were fixed in PFA 4% overnight (4 °C lightless). Cells were re-suspended in isoton buffer and analyzed by flow cytometry. Single-stain controls were used to set gating parameters and any compensation. All flow cytometry results were analyzed by FlowJo software following a rigorous doublet discrimination based on FSC-A versus FSC-H. Data were collected by the cell analyzing LSRFortessa (BD Biosciences-Immunocytometry Systems) using “BD FACSDivaTM Software” (BD Biosciences) and analyzed with “FlowJo (Tree Star) Software”.

### Wound healing assay

MDA-MB-231 and DxR/30 cells were seeded (8 × 10^5^) in 6 wells plate and let in growing conditions (DMEM 10%FBS, at 37°, 5%CO_2_) for 24 h or until cells get confluent. Then, using a 100 μl tip, a scratch was performed in the cell monolayer and cells were cultured in DMEM supplemented with 2% of FBS in order to avoid cell proliferation. To obtain the same field during the image acquisition, pen markings were performed at the bottom of the culture plates as reference points close to the scratch. Cell migration was registered by light microscopy (Evos Skedda) at 24, 48 and 72 h post scratch. Using ImageJ software the wound healing was measured and a closing-time percentage was calculated based on the initial scratch size of each cell type.

### Statistical analysis

The data were presented as mean of triplicates ± SD or as means of triplicates. Statistical significance was determined using Student’s t test, or two-way ANOVA followed by Bonferroni post-test. The criterion for statistical significance was p < 0.05.

## Supplementary Information

Supplementary Information 1.
