# Supplementary material for: Translocation of intracellular CD24 constitutes a triggering event for drug resistance in breast cancer
Source: Sci Rep. 2021 Aug 23;11:17077. doi: 10.1038/s41598-021-96449-7 (PMC8382710; doi:10.1038/s41598-021-96449-7)
Supplement: Supplementary file 1 — Supplementary Information 1. [file 41598_2021_96449_MOESM1_ESM.pdf]

Supplementary figure 1

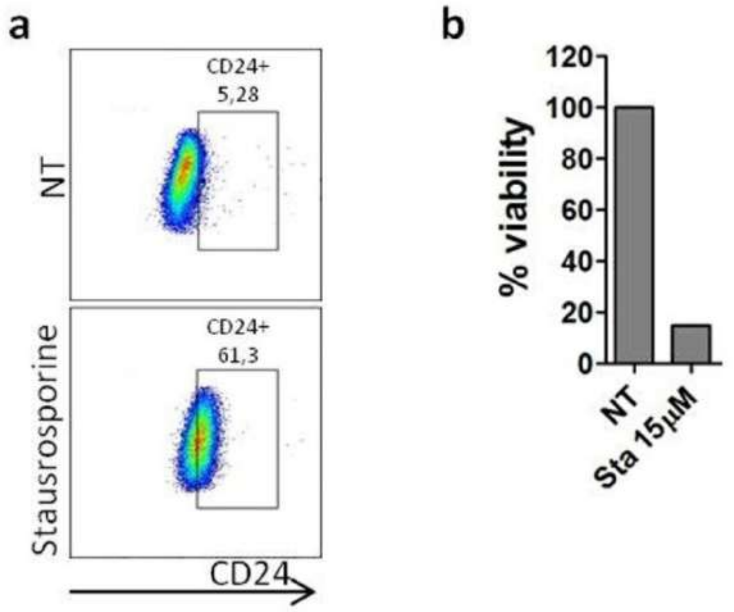

Supplementary figure 1 - Staurosporine induces CD24 translocation in MDA-MB-231 cells. **(a)** Flow cytometry analysis of CD24 cell localization after staurosporine treatment. MDA-MB-231 cells were treated with staurosporine (15µM) for 4h. Then, an extracellular staining was performed using anti-CD24/FITC. Pseudocolor plots are representative of triplicates. **(b)** MDA-MB-231 cells were treated with staurosporine (15µM) for 24h and cell viability was measured by MTT assay. Data represents means of triplicates.

Supplementary figure 2

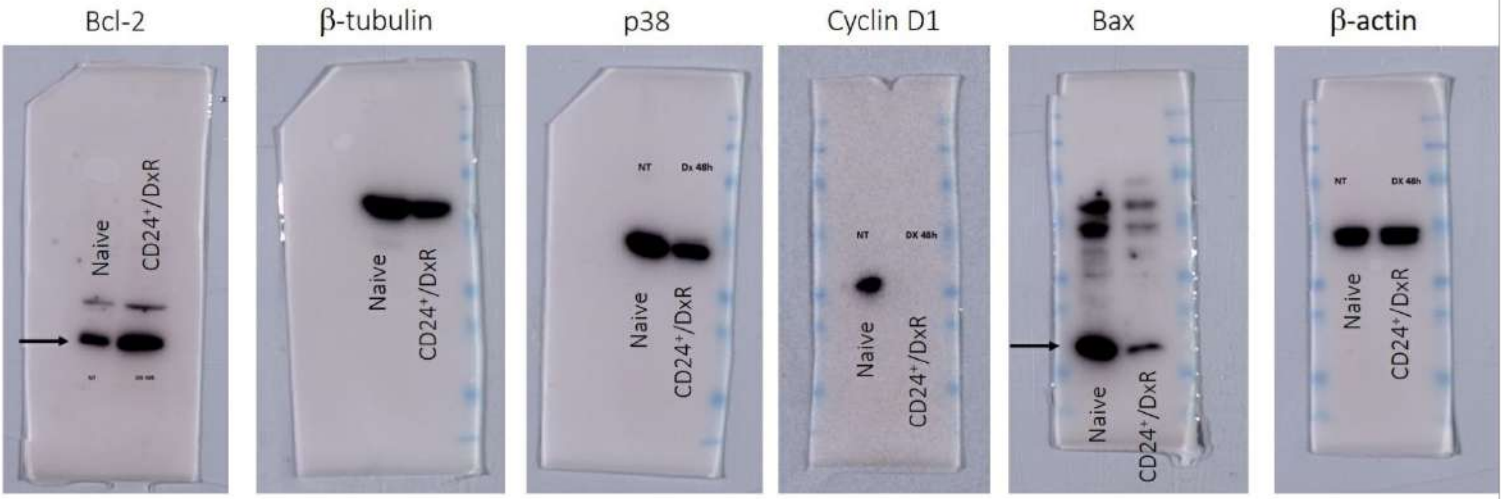

Supplementary figure 2 – Full length blots corresponding to the figure 3c of the manuscript.

Supplementary figure 3

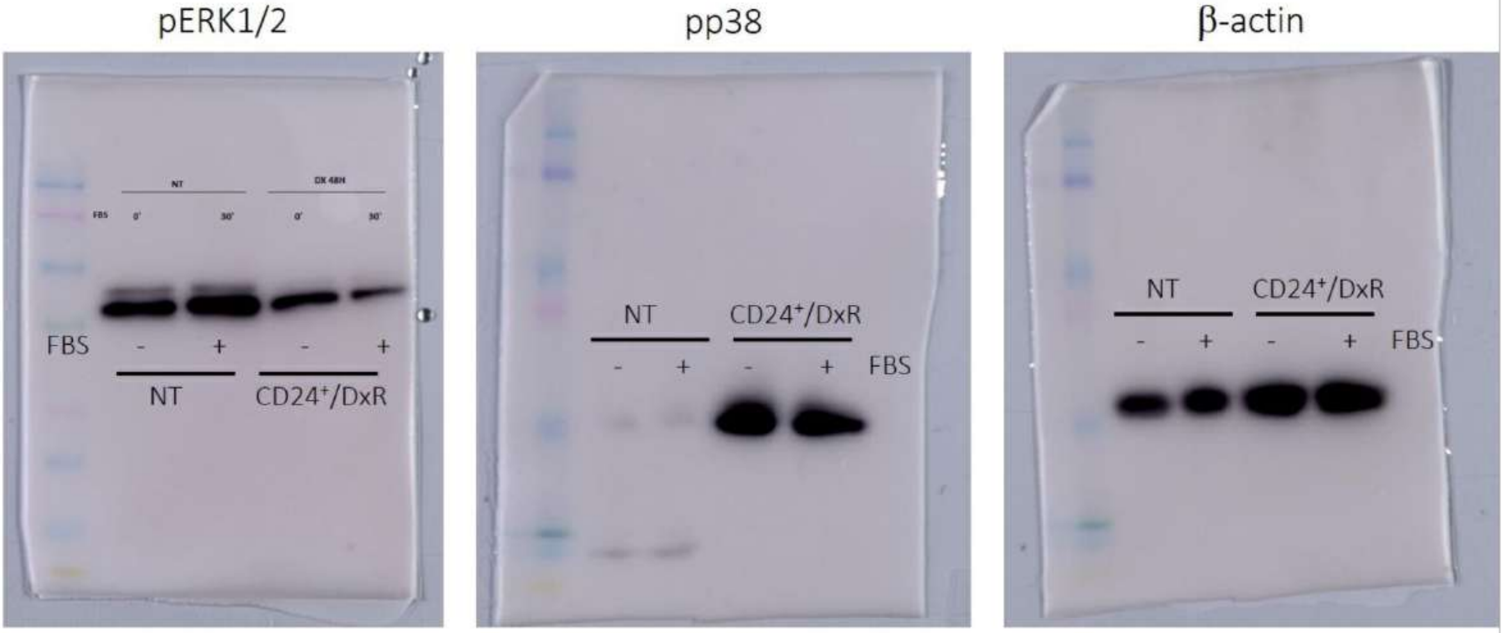

Supplementary figure 3 – Full length blots corresponding to the figure 3d of the manuscript.

Supplementary figure 4

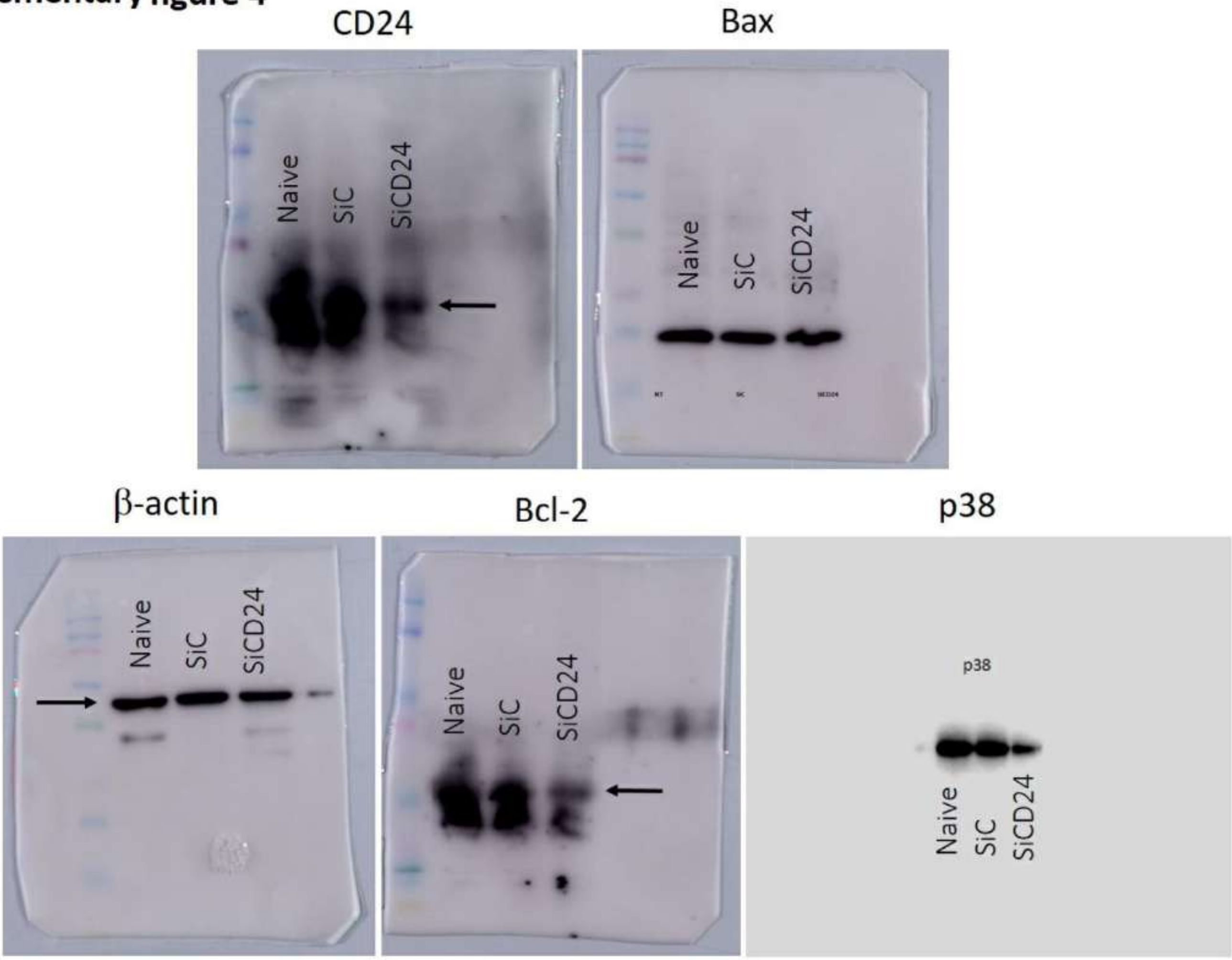

Supplementary figure 4 – Full length blots corresponding to the figure 3e of the manuscript.

Supplementary figure 4.2 (variable exposure)

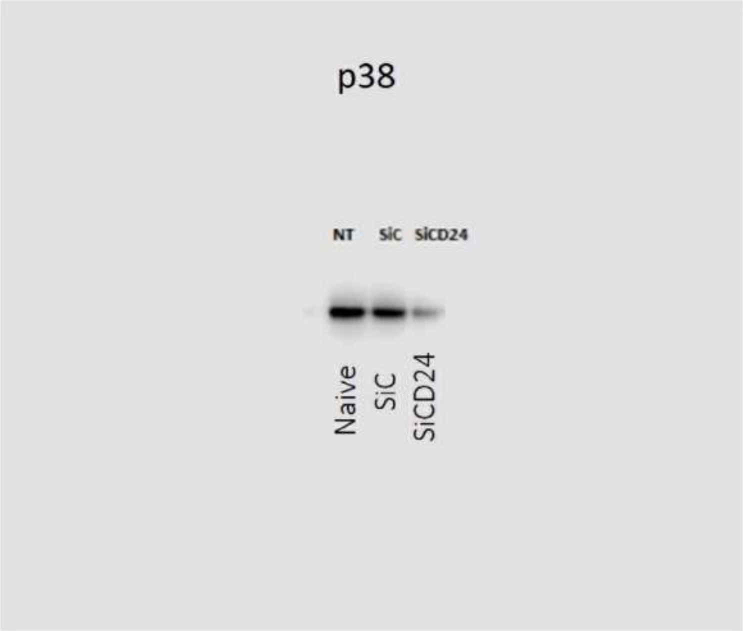

Supplementary figure 4.2 – Full length blots corresponding to the figure 3e of the manuscript.

Supplementary figure 5

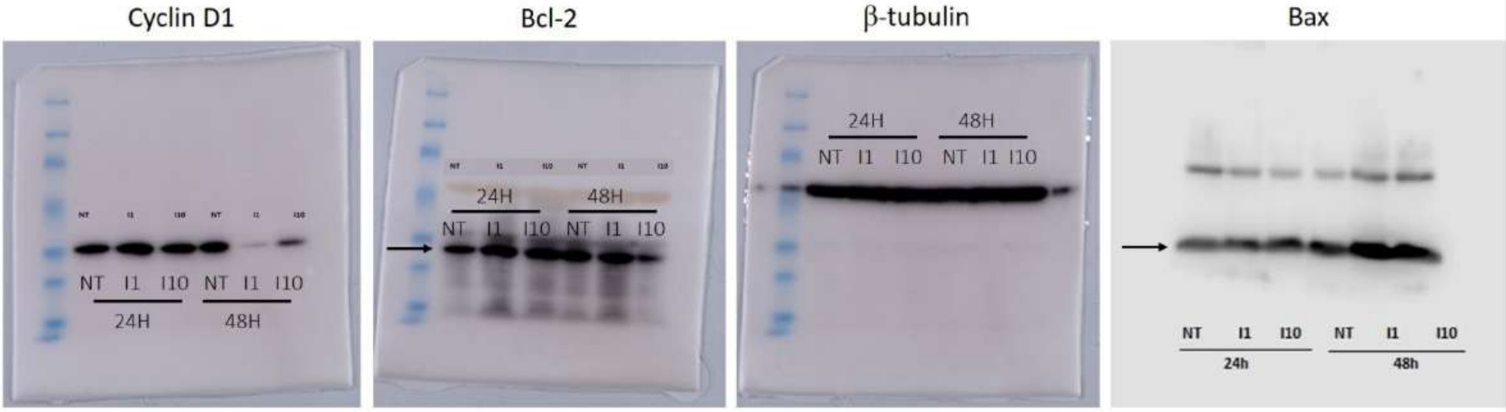

Supplementary figure 5 – Full length blots corresponding to the figure 3j of the manuscript.

Supplementary figure 6

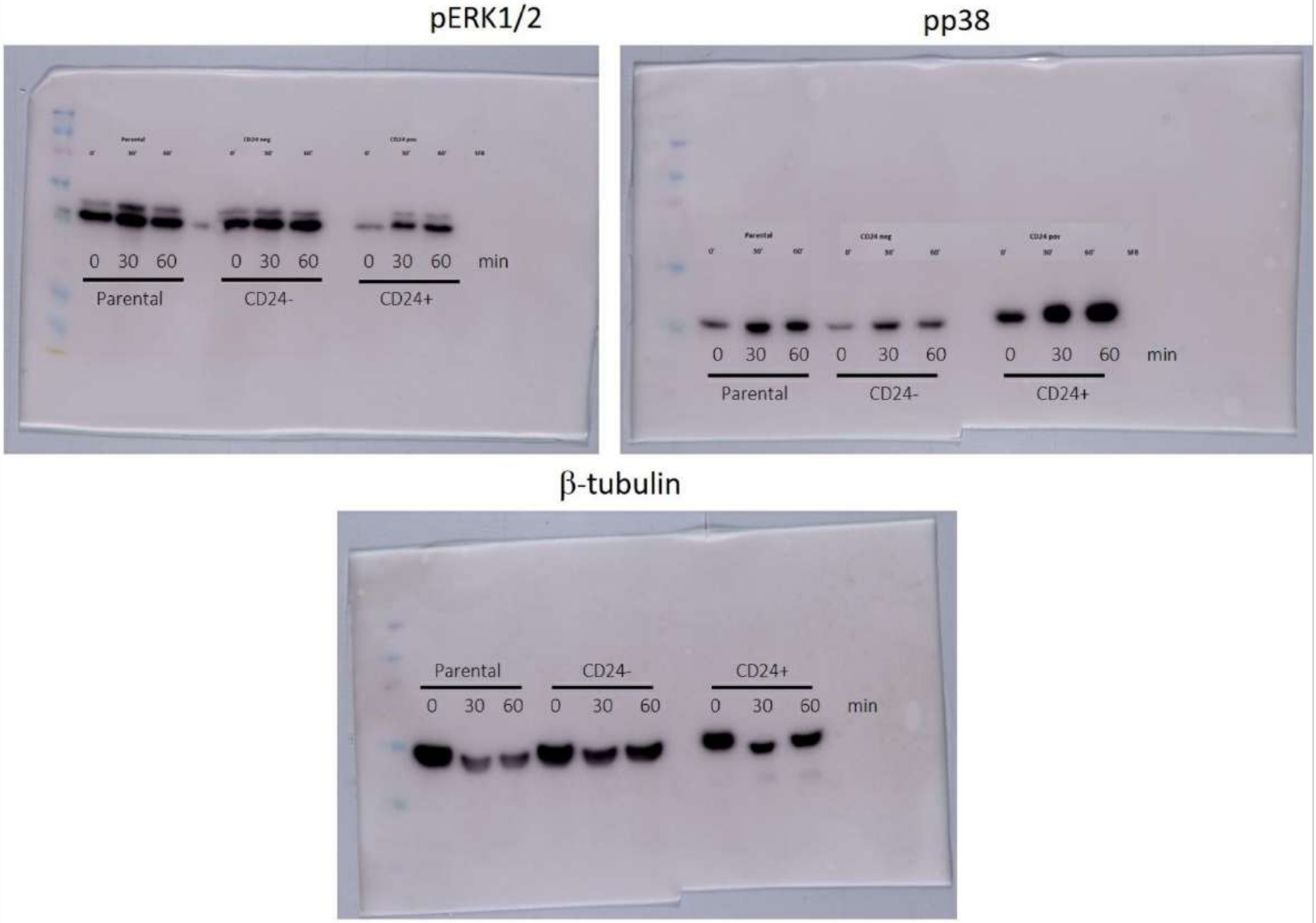

Supplementary figure 6 – Full length blots corresponding to the figure 4a of the manuscript.

Supplementary figure 7

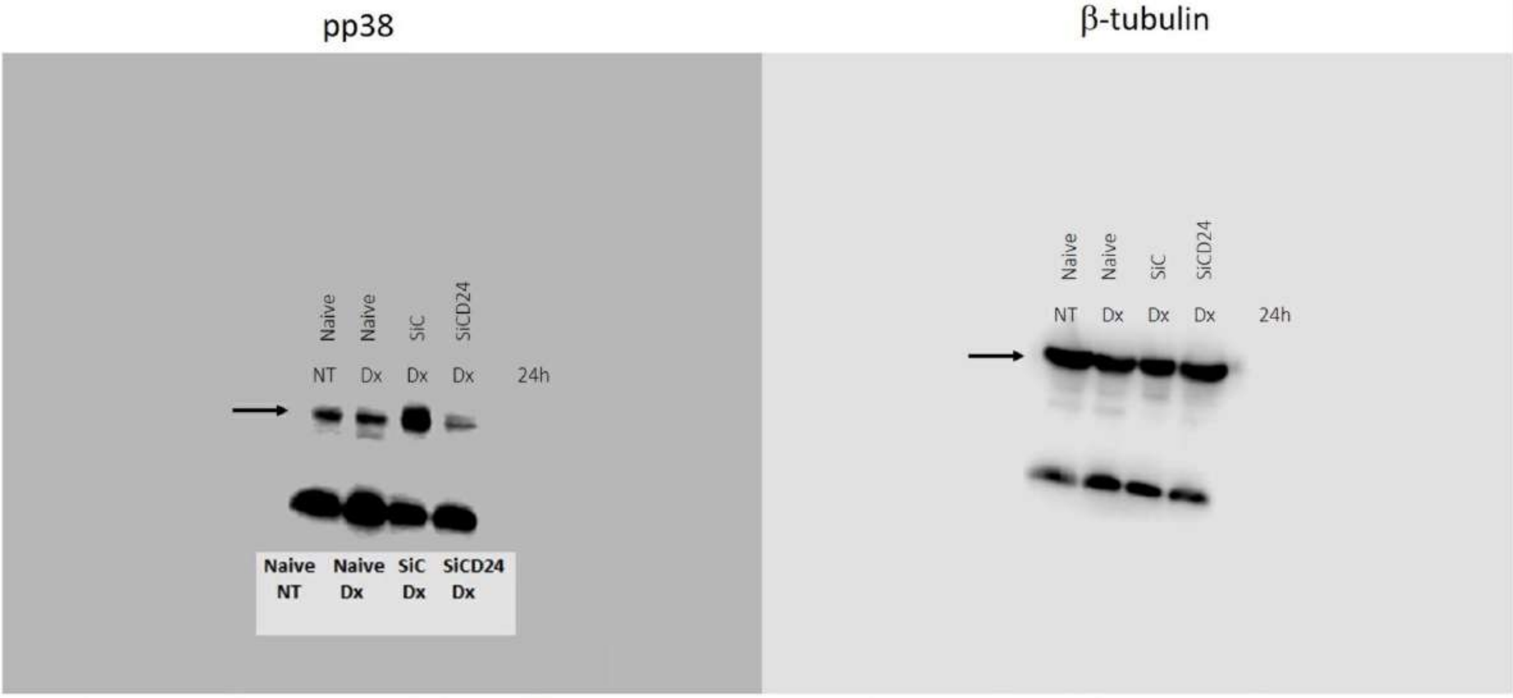

Supplementary figure 7 – Full length blots corresponding to the figure 4c of the manuscript.

Supplementary figure 8

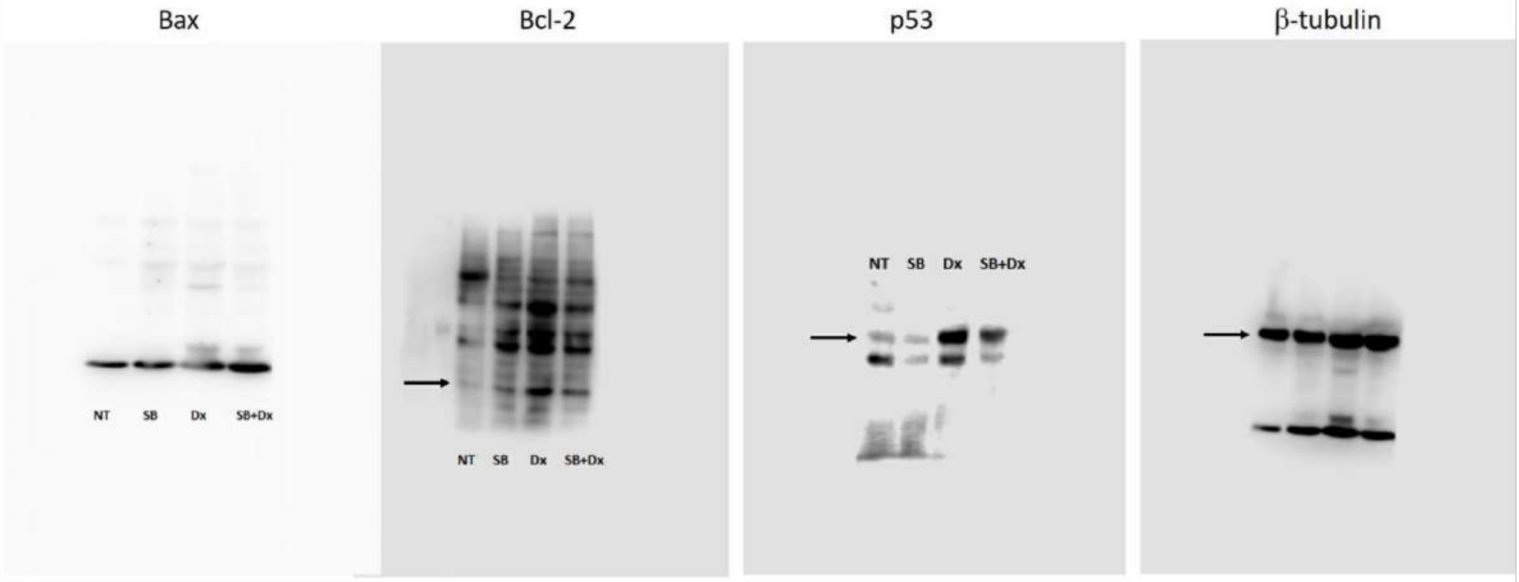

Supplementary figure 8 – Full length blots corresponding to the figure 4g of the manuscript.

Supplementary figure 9

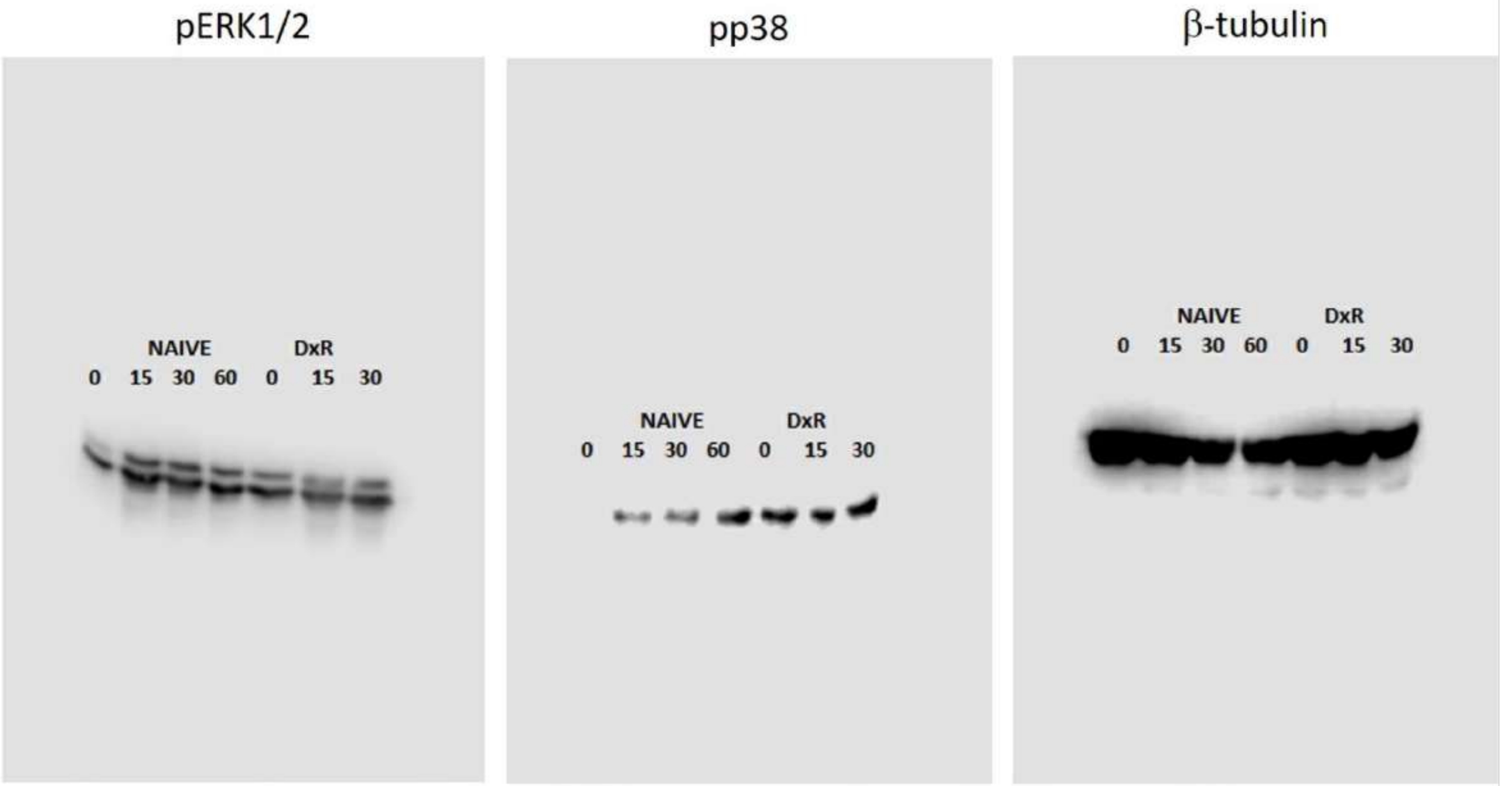

Supplementary figure 9 – Full length blots corresponding to the figure 5e of the manuscript.

Supplementary figure 9.2 (variable exposure)

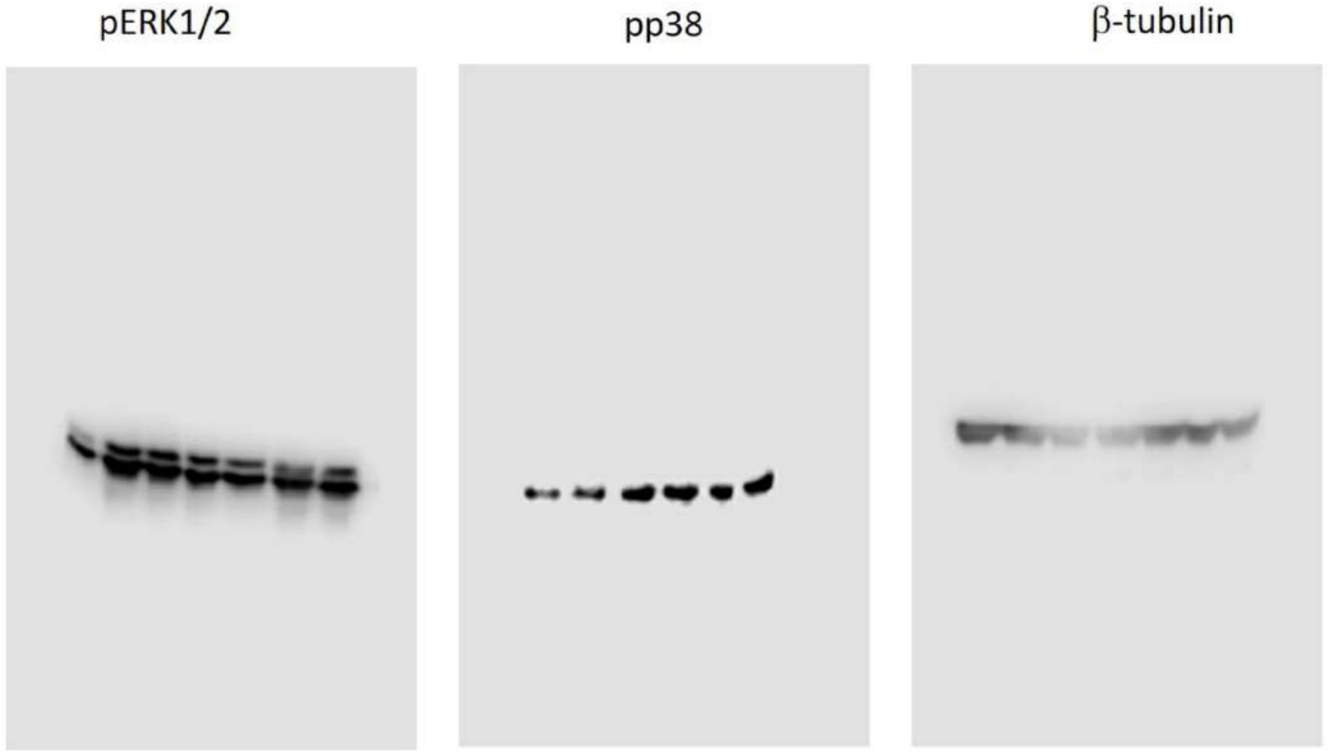

Supplementary figure 9.2 – Full length blots corresponding to the figure 5e of the manuscript.
